# Surfactant supported chitosan for efficient removal of Cr(VI) and anionic food stuff dyes from aquatic solutions

**DOI:** 10.1038/s41598-023-43034-9

**Published:** 2023-09-22

**Authors:** Magda A. Akl, Aya G. Mostafa, Magdy Y. Abdelaal, Mennat Allah K. Nour

**Affiliations:** Department of Chemistry, Faculty of Science, Mansoura University, Mansoura, 35516 Egypt

**Keywords:** Analytical chemistry, Materials chemistry, Polymer chemistry, Chemical synthesis

## Abstract

In order to develop a novel and cost-effective adsorbent with outstanding adsorption capacity and excellent recyclability for anionic pollutants, the chitosan-modified cetyltrimethylammonium bromide sorbent (CS@CTAB) was fabricated. Fourier-transform infrared spectroscopy, N_2_ adsorption–desorption isotherm, elemental analysis, Thermogravimetric analysis, X-ray diffraction, and Scanning electron microscopy have been applied to evaluate both raw and surfactant modified chitosan (CS@CTAB). Azorubine, Sunset Yellow, and hexavalent chromium were used to study the adsorption behavior of CS@CTAB under various parameters such as adsorbent dose, initial dye and metal ion concentration, contact time, and temperature. Adsorption equilibrium, kinetics models and thermodynamic parameters were investigated. The adsorption isotherm fitted well with the Langmuir isotherm model, with a maximum adsorption capacity of 492.6 mg/g, 492.6 mg/g, and 490.196 mg/g for Azorubine, Sunset Yellow, and Hexavalent Chromium, respectively. The kinetic studies showed that the pseudo-second-order model provided a better correlation between experimental data. Furthermore, the calculated thermodynamic parameters confirmed that the adsorption of Cr(VI), E110, and E122 by CS@CTAB material is a spontaneous and exothermic process. The fabricated CS@CTAB adsorbent was employed for the efficient elimination of Azorubine, Sunset Yellow, and hexavalent chromium from real water samples, synthetic mixtures, and colored soft drinks, with a percentage of recovery of ~ 96%. The plausible adsorption mechanisms of Azorubine, Sunset Yellow, and hexavalent chromium on the surface of CS@CTAB are elucidated. The adsorption anticipated to be due to electrostatic interaction and hydrogen bond formation for hexavalent chromium; while the adsorption of Azorubine and Sunset Yellow, was assumed to be due to electrostatic interaction, hydrogen bonding, and n-π interaction. Finally, the study demonstrates the efficiency of CS@CTAB for the removal of anionic species from several samples, including natural water and colored beverages.

## Introduction

Globally, water pollution is a crucial issue that concerns the human population. Large amounts of wastewater are generated daily by industrial, agricultural, and domestic activities and deposited to the land or water receptors. At the present time, the continuous discharging of effluents into water resources from industrial processes, such as textiles, rubber, cosmetics, paper, and leather, has led to the spread of toxic and carcinogenic compounds in aquatic bodies. This continuous discharging of effluents would affect human health and environmental equilibrium^1,2^. Hazardous organic and inorganic wastes such as dyes and heavy metals, have toxic effects and may even cause cancer. So, it is vital to keep the levels of both dye and heavy metals at their allowed limits in water resources^3,4^.

Synthetic dyes are used in numerous manufacturing processes as pesticides, varnish, petrochemical, electroplating, and food industries. Their discharge without any treatment leads to a series of environmental problems and growing global concerns^5,6^. These dyes are non-biodegradable in the aquatic systems because of their synthetic nature and aromatic structure. This leads to limitations of photosynthesis process as they are mutagenic substances. Therefore, it is essential to remove these substances from the discharged wastewater before reaching the receiving waterbodies^7^. Food dyes such as Sunset Yellow (E110) and Azorubine (E122) belong to the azo dye category. These dyes have excellent chemical stability, good solubility in water, high color and a complex aromatic structure. These characteristics would complicate their decay under natural conditions^8^. Sunset Yellow and Azorubine are derived from petroleum, and widely used in most food products, including juices, soft drinks, candies, jellies, and salty snacks, to give coloring^9^. The presence of these food coloring substances decreases the aesthetic value of an aquatic system, contributes to higher chemical oxygen demand (COD), and influences the passing of light^10,11^. Additionally, these dyes have harmful effects on human health as their metabolites are mutagenic, carcinogenic, and are hyperactive in children^12^.

Heavy metals are of considerable environmental concern due to their toxicity, multiple sources, nonbiodegradable properties, and accumulative behaviors. Excessive pollution of soils by heavy metals results in the metals being introduced into the food chain through plants and water. This represents a potential risk to human health^13,14^. Chromium is considered to be the most lethal heavy metal. The trivalent (III) and hexavalent (VI) forms of chromium are the two major oxidation states in which Chromium might occur. Cr(VI) is much more carcinogenic^15,16^. The industrial sources of Cr(VI) mainly include alloy and steel manufacturing, metal finishing, electroplating, leather tanning, pigments and dyeing industries. Chromium (VI) compounds, e.g. sodium chromate and potassium dichromate, are water-soluble. They exhibit adverse effects on the human respiratory system and skin. Moreover, they are found to be carcinogenic in several human organs such as the lungs, stomach, nose, and respiratory system. Also, it has aquatic toxicity^17–19^. Hexavalent chromium is nephrotoxic and tumorigenic. It has been reported in experimental animals that the hexavalent form of chromium can affect bone formation in fetal development. The mechanisms for this effect have yet to be elucidated; however, it is suggested that the potent peroxidant properties of hexavalent chromium may be involved. Animal studies have revealed a deficiency in lactation and male sterility resulting from hexavalent chromium exposure^20,21^.

Many treatment technologies are employed for the removal of pollutants from wastewater like coagulation^22^, solvent extraction^23^, ion exchange^24^, flotation^25–27^, cloud point extraction^28^, liquid–liquid extraction^29^, photocatalytic degradation^30,31^, and adsorption^32–38^. Adsorption is considered to be one of the best methods for removal of contaminants from wastewater due to its efficiency, the ease and low cost of the operation compared to other processes. A wide range of organic and inorganic adsorbents are used including zeolites, activated charcoal, clays, chitin, and polymers^39,40^.

Polymers have been regarded as a good tool to minimize the environmental impact. Among them, polysaccharide-based materials such as cellulose and its derivatives, chitosan, alginate, and carrageenan have been extensively applied as adsorbents owing to distinctive advantages such as easy availability, renewability, biodegradability, cost-effectiveness, and safety^41^.

Chitosan (CS) is a natural adsorptive polymer obtained from the deacetylation of chitin. It has an affinity towards pollutants in wastewater because it has amino (–NH_2_) and hydroxyl (–OH) groups. Chitosan is non-toxic, hydrophilic, biocompatible, and biodegradable. As chitosan molecules contain a large number of active amine (–NH_2_) groups, they have good have chelating properties and excellent adsorption capacity for both anionic dyes and heavy metal ions^42–44^. The uptake of heavy metals and dyes by chitosan has been investigated in many studies. To improve chitosan adsorption capacity, several chemical modification methods like cross-linking and new functional groups insertion, have been performed. Recently, surfactant-modified adsorbents were employed as clay, biomass, and chitosan. Consequently, surfactant modification on chitosan for the adsorption of hazardous compounds was applied^45,46^.

Surfactants are amphiphilic molecules containing two distinct parts: hydrophilic and lipophilic groups. They can be broadly defined as compounds that alter the energy relationships at interfaces, often in terms of altering either the surface or interfacial tension. Therefore, surfactants were used in the modification of many adsorbents. Examples of such surfactants include sodium dodecyl sulfate (SDS), sodium lauryl, and Cetyl trimethyl ammonium bromide (CTAB)^47^. They are used as stabilizers for adsorbents (surface modifiers) and show affinity to react with their surface.

The protonated amino groups of chitosan can produce strong electrostatic attraction with anionic species which makes chitosan a promising adsorbent for the treatment of these species. Unfortunately, chitosan has poor mechanical properties and low stability and it is too difficult to separate it from a solution which limits the application of this material in water treatment technologies. So, chitosan crosslinking and modification is so important in order to increase its capacity toward anionic species and make it an applicable material in the process of water treatment (Huo et al. 2021). CTAB surfactant is a cationic surfactant that exhibits good surface activity, antibacterial activity, and stability in both alkaline and highly acidic mediums.

Babazadeh et al. recently reported the role of surfactant modified chitosan-clinoptilolite composite in the batch and continuous adsorption systems for removal of anionic dyes (methyl orange)^48^. Furthermore, the efficiency of sodium lauryl sulfate modified chitosan in the inhibition of corrosion was investigated by Jessima et al.^49^.

In general, chemical and physical modification of adsorbents by surfactants aims to enhance the ability of these adsorbents to remove pollutants and achieve higher efficiency^50^. Cetyl trimethyl ammonium bromide (CTAB) is a cationic detergent, soluble in H_2_O, less toxic, highly biodegradable surfactant than other surfactants and has stability in both alkaline and highly acidic mediums^51^. Recently, CTAB supported organoclay has been successfully used to remove organic and inorganic pollutants from aquatic solutions [Mostafa & Akl]. Hence, CTAB is used for modification of chitosan to enhance its adsorption capacity towards adverse pollutants including anionic dyes such as (E110 and E122) and also heavy metals such as Cr(VI).

As of now, very little work has been reported on the use of CS@CTAB for the removal of anionic species like Cr(VI), Azorubine and Sunset Yellow FCF dyes from aquatic solutions.

The primary purposes of this study are:

i. Preparation of the CTAB modified chitosan.
ii. Characterization of the structural, surface functional groups and surface potential, textural, and morphological properties of CS and CTAB–modified CS with the help of various techniques viz. FTIR, N_2_ adsorption–desorption isotherm, elemental analysis, thermogravimetric analysis, XRD, and SEM.
iii. To study the optimum conditions for removal of E110, E122, and Cr(VI) by CS@CTAB like contact time, initial pH of the E110, E122, and Cr(VI) solutions, (E110, E122, and Cr(VI)) concentration, and reaction temperature.
iv. Analysis of the experimental data with various (Langmuir and Freundlich) isotherms and (pseudo-first-order and pseudo-second-order) kinetic model equations.
v. Investigating the desorption experiments using different eluents; NaOH, HCl, CH_3_COONa, ethanol, Na_2_CO_3_, and EDTA.
vi. Elucidation of the plausible mechanism of adsorption of the studied anionic species onto CS@CTAB.

## Experimental

### Materials

Chitosan (CS) was purchased from TECHNO PHARMACHEM, INDIA, with a deacetylation degree of 75%, glutaraldehyde 25% (≥ 98%), glacial acetic acid (> 99%), cetyltrimethylammonium bromide (CTAB) with a chemical formula of C_19_H_42_BrN and molecular weight of 364.46 g/mol as the surfactant. The used chemicals are NaOH, EDTA, CH_3_COONa, HCl, Ca (NO_3_)_2_, NaCl, Na_2_CO_3_, K_2_Cr_2_O_7,_ ethanol, Sunset yellow FCF (E110), and Azorubine (E122) anionic food dyes. All these chemicals were obtained from Sigma Aldrich and were analytical grade.

### Preparation of chitosan modified CTAB (CS@CTAB)

**Figure 1:**
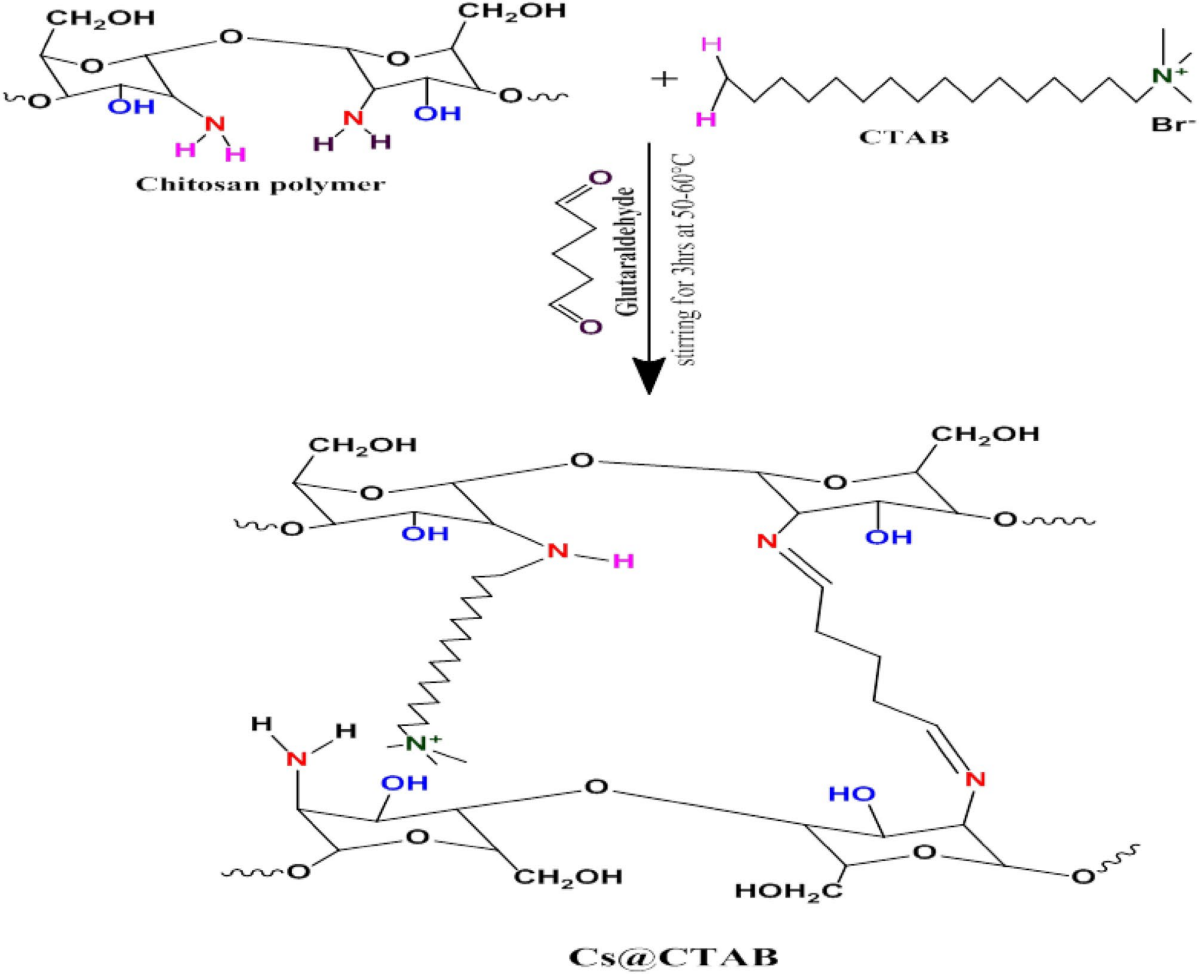
Preparation of CS@CTAB.

The synthesis of CTAB-modified CS was conducted by the following procedure^48,49^. 2 g of CS were first dissolved in 200 mL of distilled water with 2% acetic acid for 1 h at room temperature using a magnetic stirrer and then left to settle down. Afterwards, 1 g of CTAB was added with stirring for 3 h at 50–60 °C. Finally, glutaraldehyde was gradually added (equals 10% of chitosan weight) to the solution mixture and was mechanically stirred for 30 min in order to be homogenized. Eventually, the solution mixture was poured into 1 M NaOH to precipitate the prepared CS@CTAB as represented in Fig. 1. The quantity of CTAB in the CS@CTAB was calculated and it was found to be 26.26% (wt.).

### Characterization

A Perkin Elmer 550 spectrophotometer was used for the determination of the concentration of K_2_Cr_2_O_7_, anionic dyes in single and multi-components systems over a range of 200–900 nm using quartz cells. The λ_max_ was found to be 486 nm for E110, 518 nm for E122 and 427 nm for K_2_Cr_2_O_7_. The specific surface area of the CS and CS @CTAB materials was obtained using the Brunauer Emmet Teller (BET) analysis (Size Analyzer (QUANTACHROME—NOVA 2000 Series). Elemental analysis of CS, CS@CTAB was performed using a Costech (ECS-4010) elemental analyzer. Surface morphology of native CS and CS @CTAB was investigated using a JSM-6510LV model. The IR spectra of native CS and CS@CTAB, before and after adsorption of anionic species, were investigated by a Perkin-Bhaskar-Elmer Co., USA. Samples were grounded and mixed with KBr and then pressed in pellet form. X-ray diffraction (XRD) patterns of the CS and CS@CTAB materials were attained by means of PAN analytical X’Pert PRO diffractometer using the 2-theta (2θ) which ranged from 4° to 70°. The thermal stability of CS and CS@CTAB was inspected using thermogravimetric analysis (Berkin Elmer TGA 4000) on a heating rate from 30 to 900 °C of 15 °C/min. The concentrations of analytes before and after adsorption were measured spectrophotometrically using a Jasco UV–Vis spectrophotometer (model V-530, Japan). The point of zero charge (pH_PZC_) of CS@CTAB was determined as follows: 0.1 g of the CS@CTAB adsorbent was added to a 25 ml of pH-adjusted NaCl (0.01 M) solution that varied from 2 to 12 and the mixtures were allowed to shake at the equilibrated shaker for 48 h. 0.1 M of HCl and 0.1 M of NaOH were utilized for NaCl pH adjustment. After the shaking, the final pH was recorded and ΔpH was measured as in the following equation (ΔpH = pH_i_ − pH_f_) and was plotted against the initial pH (pH_i_). The pH_pzc_ value is the cross point where the curve ΔpH versus pH_i_ crosses the line ΔpH = 0.

### Adsorption and regeneration procedures

#### Batch tests

1000 ppm of stock solution of each investigated pollutant (E110, E122, and Cr(VI)) was prepared by dissolving 1.0 g of the dye or metal salt in 1000 mL of double-distilled water (DDW) to attain the required concentration. The adsorption experiments were carried out using 0.005 g of CS@CTAB in 25 mL dye solution at various experimental parameters such as concentration (50–250 ppm), pH (2.0–9.0), temperature (25–45 °C), adsorbent dose (0.005–0.03 g), contact time (15–300 min), and ionic strength. The amount of remaining pollutant in the solution was detected by UV–vis spectra at the specified wavelengths of 486, 518, and 427 nm for E110, E122, and K_2_Cr_2_O_7,_ respectively.

The removal percentage (R,%) and adsorption capacity (q_e_) of anionic species were calculated as represented in Eq. (1) and Eq. (2), respectively:

1 $$R\left( \% \right) = \frac{{C_{i} - C_{f} }}{{C_{i} }} \times 100$$

2 $$q_{e}= \frac{{\left( {C_{i} - C_{f } } \right) \times V}}{m}{ }$$

where C_f_ and C_i,_ (ppm) are the equilibrium and the initial dye concentrations, respectively. While V is the volume of analyte solution (L) and m is the weight (g) of CS@CTAB^52^.

#### Batch experiments for pollutants in the binary system

To investigate the efficiency CS@CTAB in the studied anionic species in binary systems, batch experiments were performed using 0.005 g of CS@CTAB in 25 ml of each binary system solutions with 50 ppm of each pollutant at pH 3 with shaking at 120 rpm for specified periods. Maximum adsorption of each binary systems was recorded every hour over a period of 6 h.

#### Desorption studies

Pollutants (E110 and E122) and Cr(VI) were adsorbed on the CS@CTAB surface. Then, desorption of such pollutants was investigated by applying a variety of eluents including sodium hydroxide (0.1 mol/L), Na_2_CO_3_ (1 mol/L) with and without heating, sodium acetate (0.1 mol/L), ethanol, and hydrochloric acid (0.2 mol/L). The batch technique was employed to study the regeneration of CS@CTAB over five repeated cycles of the adsorption–desorption procedure. For (Cr(VI), E110, and E122), 0.005 g of CS@CTAB was shaken in 25 ml of (100 ppm for E110, E122, and Cr(VI)) for 240 min. After that, the CS@CTAB was filtered and washed. Consequently, desorption step was carried out by utilizing 0.005 g of CS@CTAB-E110, CS@CTAB-E122, and CS@CTAB-Cr(VI) and in 25 ml Na_2_CO_3_ (1 mol/L); then shaking for 2 h at 45 °C. This process was performed for four more times. Using Eq. (3), the desorption efficiency[D,%)] of pollutants under investigation, was estimated:

3 $$Desorption \% = \frac{{amount\,\,desorbed\,\,to\,\, the\,\,solution\,\,(mg{/}l)}}{{amount\,\,adsorbed\,\,on\,\,CS@CTAB\,\,(mg{/}l)}} \times 100$$

#### Isothermal, kinetics and thermodynamics studies

##### Initial concentration and isothermal studies

Isothermal studies for E110, E122, and Cr(VI) were investigated by the addition of 0.005 g CS@CTAB adsorbent to a set of bottles containing E110, E122, and Cr(VI) solutions to be adsorbed. They initially contain between 50 and 250 ppm. For E110, E122, and Cr(VI), the latter bottles were shaken in a thermostated shaker at 150 rpm for 240 min at 25 °C. The isothermal models of Langmuir and Freundlich were used in linear form and estimated as given in Eqs. (4) and (5). A crucial parameter is the Langmuir separation factor (R_L_), which is stated in Eq. (6), is utilized to determine adsorbent-sorbate affinities. The meaning of R_L_ values can be illustrated as follows: if its value is larger than 1, it shows that the researched adsorbent is not suitable, while if its value lies between 0 and 1, it shows that the used adsorbent is suitable.

4 $$\frac{{C_{e} }}{{q_{e} }} = \frac{1}{{k_{L} q_{m} }} + \frac{{C_{e} }}{{q_{m} }}$$

5 $$\ln q_{e} = \ln K_{f} + \frac{1}{n} ln C_{e}$$

6 $$R_{l } = \frac{1}{{1 + K_{l} C_{o} }}$$

As, C_e_ (ppm) is the concentration of pollutant (E110, E122, and Cr(VI)) at equilibrium, q_e_ (mg/g) is the adsorption capacity of pollutant at equilibrium, q_m_ (mg/g) adsorption maximum amount, 1/n is the heterogeneity factor, while K_L_ (L/mg), K_F_ (mg/g), and K are Langmuir, Freundlich constants.

Two kinetic models, pseudo-1st-order and pseudo-2nd-order, given in (Eqs. (7) and (8) respectively, were used in kinetic studies for the evaluation of the adsorption rate-limiting step. The experiments were carried out utilizing 25 ml (100 ppm of E110, E122, and Cr(VI)) and 0.005 g of CS@CTAB at the optimum pH for each pollutant. The mixture was shaken at room temperature with a constant speed of 150 rpm for different contact times ranging from 15 to 300 min.

7 $$\frac{1}{{q_{t} }} = \frac{{K_{1} }}{{q_{{e^{t} }} }} + \frac{1}{{q_{e} }}$$

8 $$\frac{t}{{q_{t} }} = \frac{1}{{K_{2} q_{{e^{2} }} }} + \frac{t}{{q_{e} }}$$

The adsorption efficiency for examined pollutants at equilibrium and at a specific time t (min) are stated as q_e_ (mg/g) and q_t_ (mg/g), respectively. Besides K_1_ and K_2_ are constants for pseudo-1st-order and pseudo-2nd-order, sequentially.

##### Temperature and thermodynamics

In a balanced shaker running at a constant speed of 150 rpm for 240 min, a set of 50 ml stoppered bottles holding 25 ml of pollutant solution (100 ppm) for [E110, E122, and Cr(VI)], 0.005 g of CS@CTAB at varying temperatures (298–318 K) and ideal pH value for each pollutant, were shaken. After adsorption and filtering, the concentration of remaining (E110, E112, and Cr(VI)) was detected. The following equations (Eqs. (9) and (10) describe the thermodynamic parameters viz. adsorption enthalpy (ΔH^o^), free energy (ΔG^o^), and entropy (ΔS^o^)); ΔS^o^ and ΔH^o^ were estimated from intercept of Eq. (10), which equals ΔS^o^/R, and slope, which equals -ΔH^o^/R of Ln K_c_ vs. 1/T. The universal gas constant (R) is equivalent to 8.314 J/mol K.

9 $$\Delta G^{o} = { } - RT\ln K_{c}$$

10 $$\ln K_{c} = \frac{{\Delta S^{o} }}{R} - \frac{{\Delta H^{o} }}{RT}$$

#### Applications in real samples

##### Natural water samples

To investigate the applicability of CS@CTAB, different water samples including (Tap and seawater) were employed for the adsorption of E110, E122, and Cr(VI) in (100 ppm). In advance of the spiking of the pollutants, the natural water samples were digested by the addition of 0.5 g of K_2_S_2_O_8_ and 5 ml H_2_SO_4_ 98% (w/w) to 1000 ml of water sample and heated for 120 min at 90 °C for complete digestion of organic materials. After cooling to room temperature, 0.005 g of CS@CTAB was introduced to these samples, and the appropriate pH value for each pollutant was set with continuous shaking for 240 min for E110, E122, and Cr(VI). The solutions were centrifuged and another 0.005 g of CS@CTAB was introduced to the supernatant to guarantee the complete separation of analytes. The remaining of each of E110, E122 and Cr(VI) was determined using a Unicam UV 2100 UV/Visible spectrometer at appropriate wavelengths.

##### Industrial samples and colored soft drinks

In order to remove E110 and E122 from degassed carbonated beverages and jelly, CS@CTAB was utilized. Initially, the carbonated beverages (an orange beverage with E110 dye and a pomegranate beverage with E122 dye) were degassed by leaving them in the air at 25 °C for 120 min. After being digested (which means the breakdown of unwanted substances in the sample) using acetic acid (4%), the strawberry and orange-flavored jelly was dissolved in DDW^53,54^. For E110 and E122, CS@CTAB (0.005 g) was introduced to each sample, and the optimum pH for each dye was set with constant shaking for 240 min. Using the Unicam UV 2100 UV/Visible spectrometer at the proper wavelengths, the residual E110 and E122 was identified.

## Result and discussion

### Materials' design

**Figure 2:**
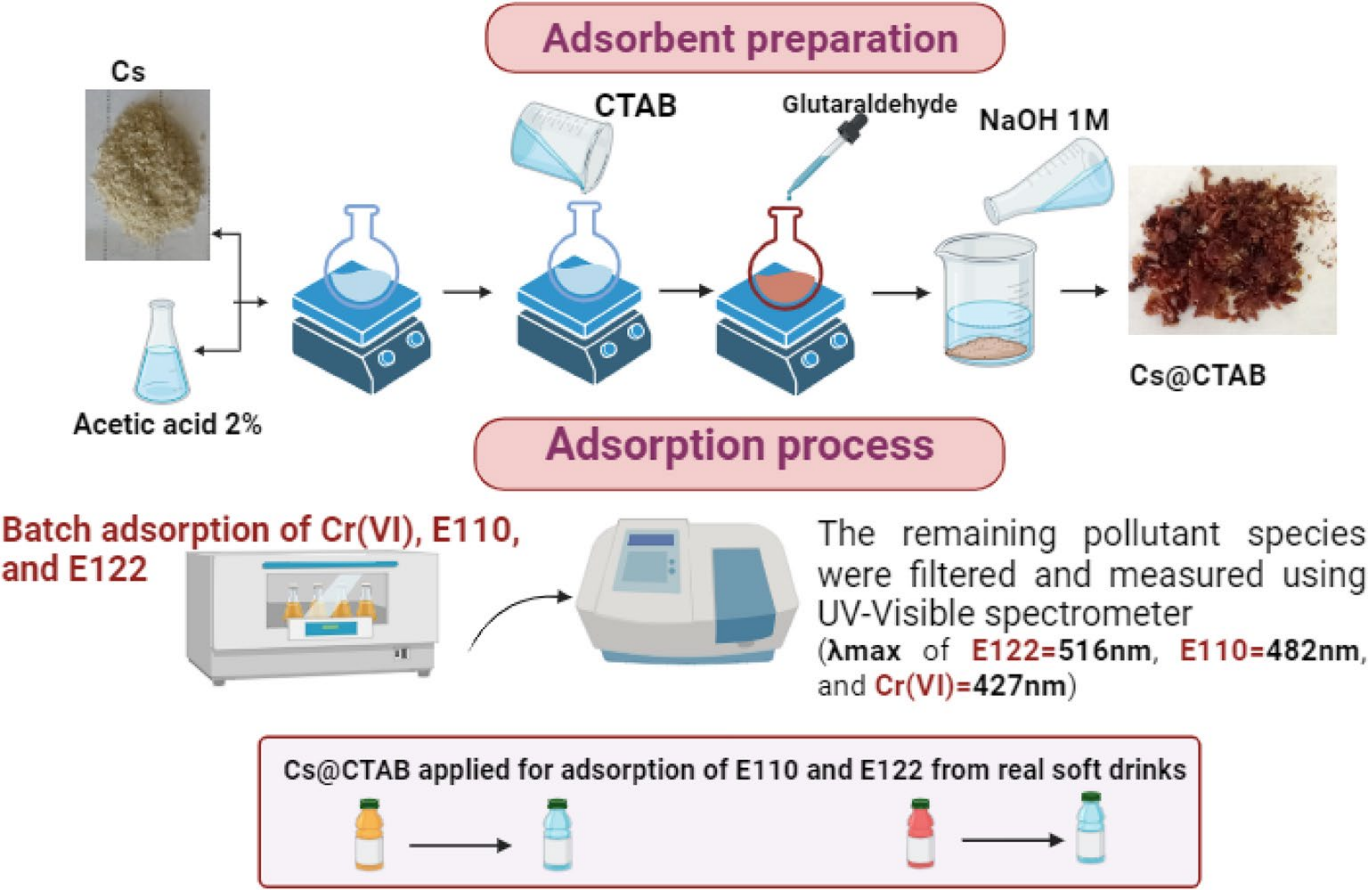
Systematic steps for adsorption technique.

Figure 2 represents the CS@CTAB synthesis steps, adsorption of the three investigated anionic pollutants and their determination. Moreover, the application of the prepared CS@CTAB on real (soft drinks) samples is included.

### Characterization

#### N_2_ adsorption/desorption isotherm Brunauer–Emmett–Teller (BET) analysis

**Figure 3:**
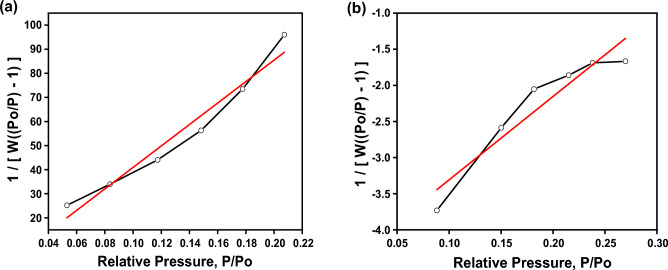
BET of: (**a**) Chitosan, and (**b**) CS@CTAB.

The surface area of the pure chitosan and CS@CTAB were analyzed using the BET method, Fig. 3. The results, presented in Table 1, show that the BET surface area of pure chitosan increased from 7.876 to 492.133 m^2^/g in CS@CTAB. The increase in the surface area of an adsorbent leads to enhancing the chitosan adsorption capacity for the pollutants.

**Table 1:** BET analysis of CS and CS@CTAB.

Adsorbent	SBET (m^2^/gm)
CS	7.876
CS@CTAB	492.133

#### Elemental analysis

The elemental analysis was evaluated for CS and CS@CTAB materials to prove the modification step. The results, presented in Table 2, show a decrease in the nitrogen percentage from 7.5 to 6.3606% in the CS@CTAB sample with an increase in carbon atom percentage from 44.1% to 57.0178% and hydrogen atom percentage from 6.0377 to 9.1308%. The CTAB surfactant, with chemical formula C_16_H_33_N(CH_3_)_3_, its reaction with the chitosan resulted in the insertion of a long carbon chain with one nitrogen group, which led to a decrease in the nitrogen percentage in the modified material. So, these results prove the formation of the CS@CTAB material by reacting chitosan with CTAB in the presence of glutaraldehyde.

**Table 2:** Elemental analysis of CS and CS@CTAB.

Sample	C (%)	H (%)	N (%)
CS	44.1	6.0377	7.5
CS@CTAB	57.0178	9.1308	6.3606

#### Morphology

**Figure 4:**
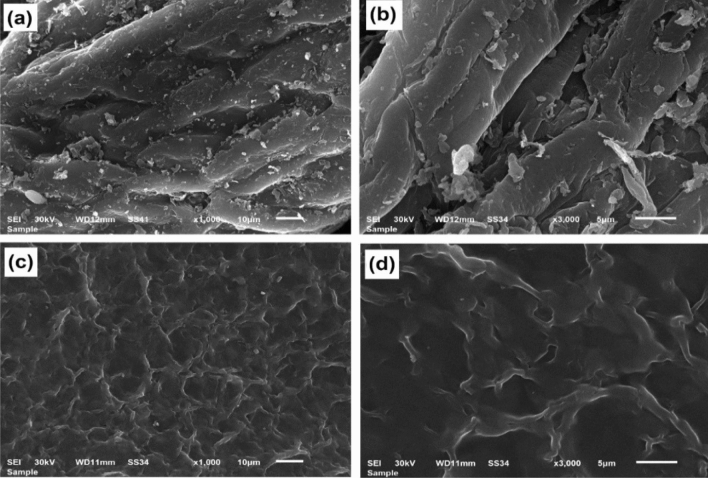
SEM images of: (**a**, **b**) Chitosan and (**c**, **d**) CS@CTAB.

Figure 4 demonstrates SEM micrographs for CS (Fig. 4a,b) and CS@CTAB (Fig. 4c,d). These micrographs show that CS@CTAB has a dissimilar morphology to native CS. SEM micrographs of CS show smooth and regular surface topology, while CS@CTAB offers a much rougher and irregular surface structure. The SEM outcomes prove that the modification of CS increased the number of pores and produced a rougher surface. Based on SEM micrographs, the aromatic rings on the CS structure cause adverse surface topology along with high porosity and surface roughness when compared to native CS^55^.

#### FT-IR spectra

**Figure 5:**
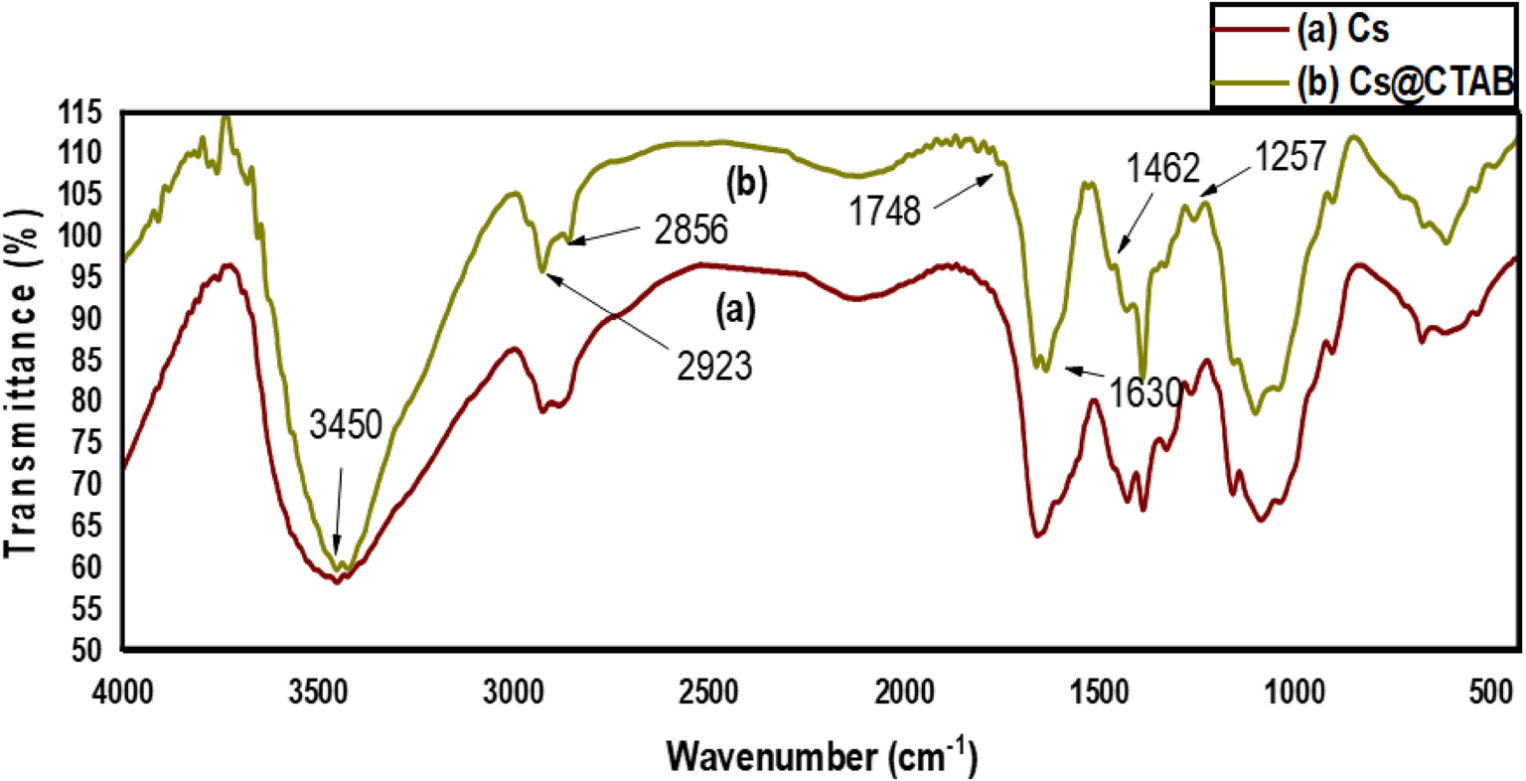
IR spectra of: (**a**) Chitosan, and (**b**) CS@CTAB.

The IR spectrum of CS, Fig. 5a, demonstrates a broad peak centered at 3450 cm^−1^ assigned to the stretching vibration of the -OH, -NH_2_ groups and to intramolecular and intermolecular hydrogen bonding. The peaks at 2930 cm^−1^, 2884 cm^−1^, 1427 cm^−1^, 1381 cm^−1^, and 1321 cm^−1^ are due to stretching and bending vibration of C–H of –CH_3_, and –CH_2_ groups. The peak at 1653 cm^−1^ is due to stretching vibration of –C=O in –NHCO, whereas the peak at 1603 cm^−1^ is due to -NH bending vibration of NH_2_. Other peaks at 1155 cm^−1^, and 1090 cm^−1^ correspond to the –C–O bending.

On the other hand, the IR spectrum of CS@CTAB, Fig. 5b, shows the following peaks: a peak at 3450 cm^−1^ appears to be less broad and sharper due to the reaction of the –NH_2_ group with CTAB and glutaraldehyde so this peak only describes the –OH group. The peaks at 2856 cm^−1^ and 2923 cm^−1^ are due to the –CH groups, those peaks increased in intensity and sharpness due to the increase in –CH_2_ groups as a result of introducing CTAB molecule which contains 16 additional –CH_2_ groups. Whereas the peak at 1748 cm^-1^ is due to –C=N that appeared as a result of addition of glutaraldehyde. The peak at 1462 cm^−1^ appeared to be due to the presence of -CH alkane groups introduced by the addition of CTAB surfactant. The other peak at 1257 cm^−1^ is due to the –C–N amine interaction^56^.

#### Thermogravimetric analysis (TGA)

**Figure 6:**
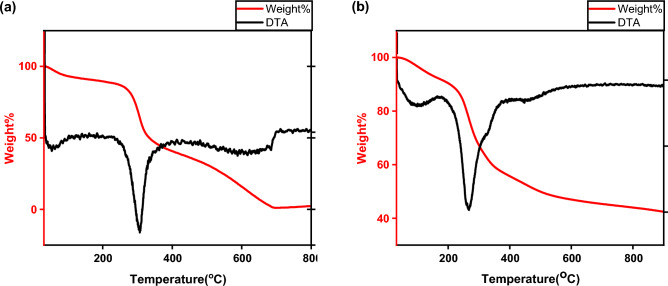
Thermal gravimetric analysis of: (**a**) Raw chitosan, (**b**) CS@CTAB.

Figure 6 reveals the several stages of weight loss characteristic of CS; the initial one was between 35 and 100 °C denoting the dehydration of the polymer. The subsequent stage was between 235 and 400 °C, which is characteristic of the breakage of the CS chain, depolymerization, and decomposition of the acetylated and deacetylated parts. The last phase was between 410 and 710 °C; marking the degradation of the products created via the preceding incident^57–59^.

The chief loss presented at temperatures of 175 °C to 400 °C was due to the decomposition of fresh CTAB (which is around 240%) and the liberation of boundless water^60–62^.

#### X-ray diffraction analysis (XRD)

**Figure 7:**
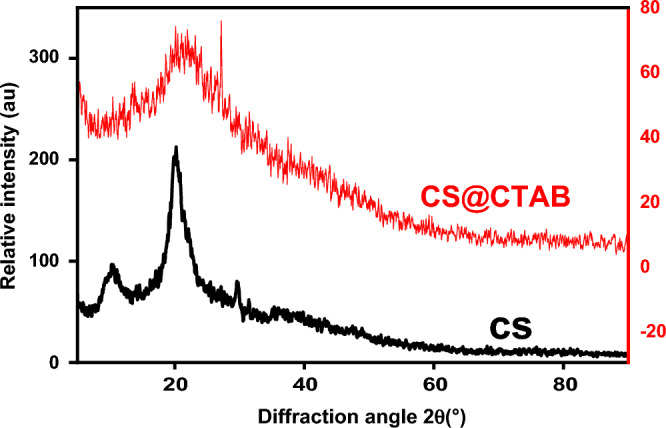
XRD patterns of: CS and CS@CTAB samples.

Figure 7 shows the XRD of both CS and CS@CTAB. The XRD of CS reveals that CS has a low-crystalline composition that can be established via two peaks: one at 10.26°, which is linked to the hydrated low-crystalline composition, and the other at 20.165°, which is a result of the amorphous state of the material. These peaks were found to be distinctive to the raw CS diffractogram; they also suggested the formation of intra- and intermolecular hydrogen bonding in addition to the existence of the free NH_2_ groups in the raw CS composition^61^.

The XRD of CS@CTAB displayed no considerable diffraction peak in XRD, with the exception of that at 20.885°. Consequently, CS@CTAB displays amorphous features that are dissimilar to those of its main constituents^60^.

Scherrer’s equation is represented in Eq. (11):

11 $$D = \frac{K\lambda }{{\beta \cos \theta }}$$

where D is the nano crystallite size. K is a constant related to crystallite shape, normally taken as 0.9. β is the peak width of the diffraction peak profile at half maximum height (FWHM) resulting from small crystallite size in radians. *λ* is the X-ray wavelength in nanometer (nm).

By calculating the value of D for both raw and modified chitosan the values were found to be 1.734 and 17.23, respectively^63^.

### Adsorption studies

#### Point of zero charge (pH_PZC_)

**Figure 8:**
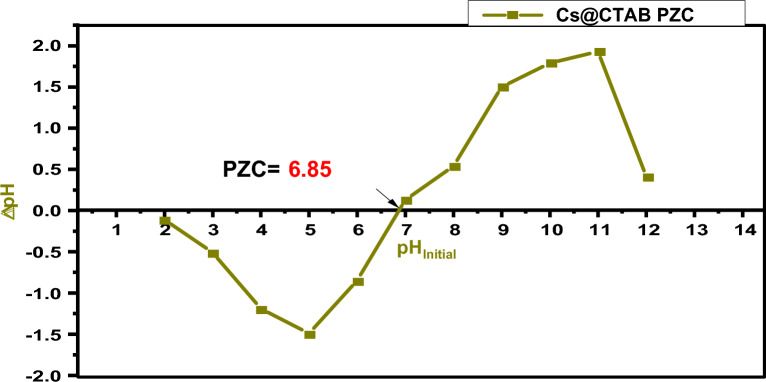
Point of zero charge of CS@CTAB.

The point of zero charge (pH_pzc_) is generally described as the pH at which the net charge of the adsorbent's surface is equal to zero in an aqueous solution during the adsorption of ionic species. In this study, a plot for pH_pzc_ determination of CS@CTAB is shown in Fig. 8. The CS@CTAB (pH_pzc_) has been attained by applying the previously published studies. Figure 8 demonstrates the variation of ΔpH value (pH_initial__pH_final_) of CS@CTAB as a function of the pH_initial_. The value of the pH_pzc_ of CS@CTAB is found to be 6.85. This illustrates that at pH less than 6.85, the CS@CTAB surface is considered to have positive charges^64^.

#### Effect of pH

**Figure 9:**
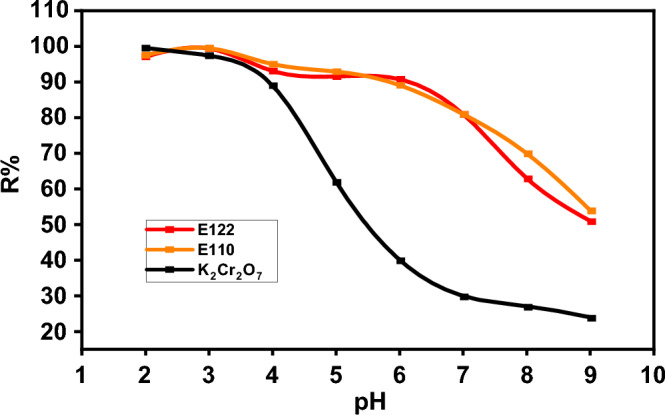
pH effect on E122, E110, and Cr(VI) adsorption efficiency on CS@CTAB under optimum conditions (temp.: (25 °C), dose: 0.005 g., conc.: 100 ppm for E110 & E122 and Cr(VI) in 25 ml volume).

The pH effect, which is demonstrated in Fig. 9, is a crucial factor in the removal of environmental pollutants. The effect of pH was carried out by varying the pH in the range of 2.0–9.0 keeping other parameters constant (shaking for 4h, at room temperature, and using a concentration of 100 ppm). It is expected that treatment of CS with the CTAB will lead to the formation of potentially several nitrogen moieties on the CS surface^65^. Elemental composition evidence (Table 1) indicates a nitrogen of 6.36% (wt%) on the CS@CTAB material, most likely indicating the formation of a variety of more basic nitrogen moieties on the CS@CTAB surface. Nitrogen-based surface functionalities can be seen with the presence of a broad FTIR peak with the midpoint at approximately 3450 cm^−1^ potentially indicating the presence of a basic amide surface functionality^65^. Following the adsorption process in the current work, in the CS@CTAB FTIR spectrum after (E122, E110, and Cr(VI)) adsorption the specific peak at 1650 cm^−1^ disappeared for the three pollutants accompanied by the appearance of S–O and S=O specific peaks for the CS@CTAB-E110 and C@CTAB-E122.

Based on experimental data, it was found that the maximum removal efficiency (R %) of Cr(VI) was 99.5 at pH 2 and 99.5 for E110 and E122 at pH 3, but decreased sharply and converged as the solution pH increased (Fig. 9). This is consistent with other batch studies which also reported maximum Cr(VI) adsorption in acidic environments^37,66,67^ and can be explained by considering the surface charges of the adsorbents and the Cr(VI) adsorbate species^68^. The surface charge of an adsorbent is determined by pHpzc where an adsorbent surface is neutral at pH = pHpzc, but develops a positive charge at pH < pHpzc and a negative charge at pH > pHpzc. Therefore at pH 2 CS@CTAB had positive surface charges; however, as solution pH increased, the positive charges are reduced and became negative^69^ once the pH values increased above its pHpzc (pH 6.85), (Fig. 8). In addition, the main ionic forms of chromate at acidic pH (pH 1.0–6.8) are hydrogen chromate (HCrO_4_^–^) and dichromate (Cr_2_O_7_^2−^) with chromate (CrO_4_^2−^) being predominant at pH > 6.8 and this results in an electrostatic attraction to the positively charged groups present on the adsorbent surface. In contrast, increasing solution pH led to a decrease in the Cr(VI) uptake due to the deprotonation of surface functional groups, along with competition between the Cr(VI) ions and the OH^−^ ions to occupy the active sites. Given the enhanced adsorption of Cr(VI) onto CS@CTAB at pH 2 (Fig. 3A), the remainder of this study focused on Cr(VI) removal by CS@CTAB using a pH 2 solution as optimal. Based on the evidence, it appears that the principal binding process is one of an electrostatic interaction between the sulfonyl moieties and the binding Cr(VI) species.

Because of the strong protonation, the adsorbent surface becomes positively charged at low pH. The Cr(VI) adsorption was enhanced due to the electrostatic force between negatively charged HCrO_4_^−^ and Cr_2_O_7_^–2^ and SO_3_^−^ of (E110 and E122) and the positively charged adsorbent surface^37^. Thus, this, in turn, enhances the affinity of CS@CTAB material towards attracting positively charged metal ions and dye molecules depending on pH control, causing the improvement of Cr(VI), E110, and E122 adsorption as shown in the equations below (12) and (13), respectively.

12 $${\text{CS}}@{\text{CTAB}}^{ + } + \left( {{\text{E11}}0/{\text{ E122}}} \right) - {\text{SO}}_{{3}}^{ - } \leftrightarrow {\text{CS}}@{\text{CTAB}}^{ + } \ldots {\text{SO}}_{{3}}^{ - } \left( {{\text{E11}}0{\text{/ E122}}} \right)$$

13 $${\text{CS}}@{\text{CTAB}}^{ + } + \, \left( {{\text{Cr}}\left( {{\text{VI}}} \right)} \right) - {\text{O}}^{ - } \leftrightarrow {\text{CS}}@{\text{CTAB}}^{ + } \ldots {\text{Cr}}\left( {{\text{VI}}} \right) \, \left( {{\text{K}}_{{2}} {\text{Cr}}_{{2}} {\text{O}}_{{7}} } \right)$$

These results are consistent with other studies presented^37,70–72^.

#### Effect of dose

**Figure 10:**
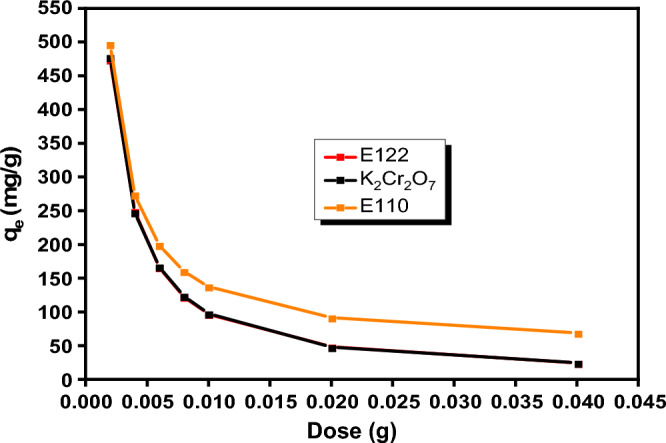
Effect of dose on E122 (100 ppm), E110 (100 ppm), and Cr(VI) (100 ppm) adsorption on CS@CTAB at room temperature for 4 h.

The influence of dose of CS@CTAB, Fig. 10, was examined in the range of (0.005–0.01 g), keeping the other parameters constant (shaking for 4 h, at room temperature, using a 25 ml of 100 ppm (E110, E122, and Cr(VI)) and an appropriate pH value for each pollutant). The maximum adsorption capacity for all pollutants took place using 0.005 g of CS@CTAB in 25 ml pollutant.

#### Effect of initial concentration

**Figure 11:**
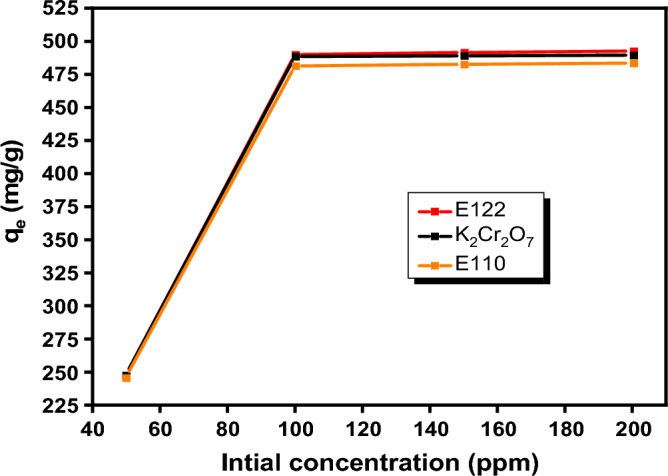
Effect of initial concentration of E110, E122 and Cr(VI) on adsorption.

The effect of the initial concentrations of pollutants on the adsorption of the studied analytes, was investigated in the range of (50-200 ppm) as shown in Fig. 11, using 0.005 g CS@CTAB adsorbent and appropriate pH value for each pollutant while keeping the other parameters constant (shaking for 4 h, at room temperature). From the graph, the adsorption capacity of CS@CTAB increases with the increase in the initial concentration of pollutants and then becomes constant at higher concentration values. The adsorption capacity increased with initial pollutant concentration due to availability of large number of binding sites of the surface of CS@CTAB. At high pollutant concentrations, the adsorption capacity reached maximum value suggesting that the binding sites on the CS@CTAB surface reached saturation.

#### Adsorption isotherm

**Figure 12:**
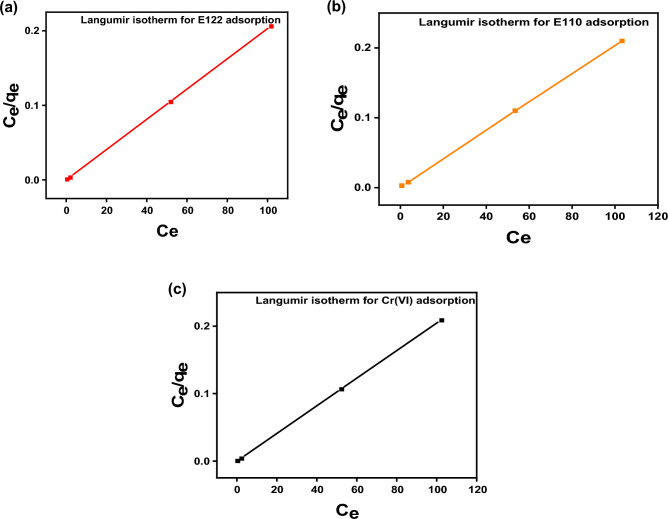
Langmuir isotherm of (**a**) Langmuir isotherm model E122 adsorption, (**b**) E Langmuir isotherm model 110 adsorption, and (**c**) Langmuir isotherm model Cr(VI) adsorption.

**Figure 13:**
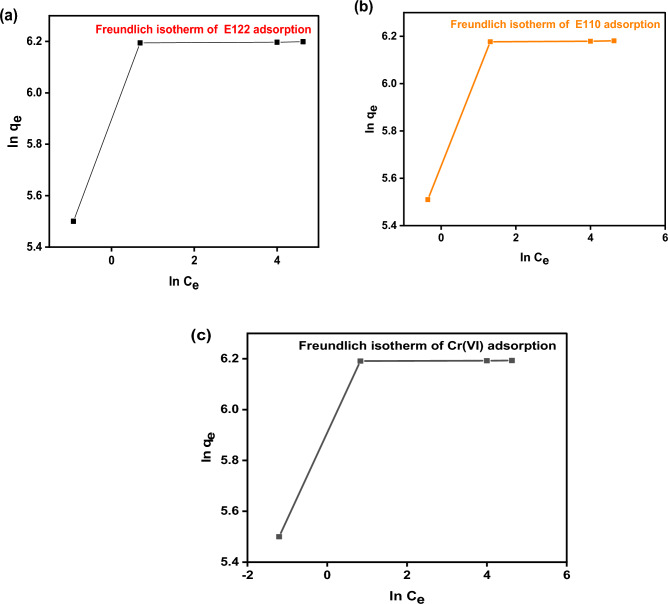
(**a**) Freundlich isotherm of E122 adsorption, (**b**) Freundlich isotherm of E110 adsorption, and (**c**) Freundlich isotherm of Cr(VI) adsorption.

The Langmuir and Freundlich isotherm are presented in Fig. 12 and 13 and the calculated coefficients are shown in Table 3. The Langmuir isotherm model (R^2^ = 0.999) seemed to describe better the adsorption process of Cr(VI), E110, and E122 by the CS@CTAB than the Freundlich isotherm model (R^2^(Cr(VI) = 0.543, R^2^(E110) = 0.962, and R^2^(E122) = 0.5435). As can be noticed, the Langmuir model accurately fits the adsorption of E110, E122, and Cr(VI) on CS@CTAB. This shows that the Cr(VI), E110, and E122 molecules bind to the active sites on the biosorbent surface as a monolayer^31^. The R_L_ values obtained for all initial concentrations of Cr(VI), E122, and E110 lie between 0 and 1 (Table 3), indicating that biosorption of Cr(VI) ions and the two dyes by CS@CTAB is a favorable process.

**Table 3:** Langmuir and Freundlich isotherm constants and correlation coefficients of CS@CTAB film for the three pollutants:

Adsorbents	Langmuir isotherm constants			
	K_L_(L/g)	q_m_(mg/g)	R^2^	R_L_
CS@CTAB- E122	5.2	492.6	0.99998	0.00192
CS@CTAB- E110	2.137	492.6	0.99994	0.00466
CS@CTAB- Cr(VI)	6.897	490.196	0.99999	0.00145

Adsorbents	Freundlich isotherm constants			
	K_F_	n	R^2^	
CS@CTAB-E122	389.68	3.57	0.53752	
CS@CTAB-E110	442.22	4.91	0.96275	
CS@CTAB-Cr(VI)	382.32	2.87	0.54352	

#### Effect of oscillation time and adsorption kinetics

**Figure 14:**
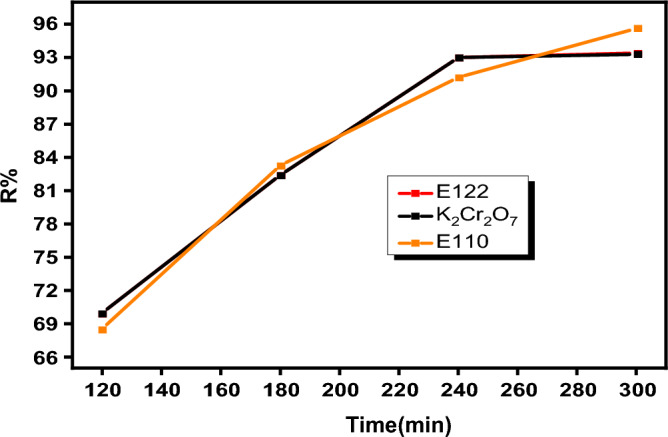
Effect of time on E110, E122, and Cr(VI) adsorption on CS@CTAB adsorbent.

For the investigation of reaction time influence on the adsorption of pollutants, the time was changed in the range of (30-300 min) using 0.005 g CS@CTAB and appropriate amount for each pollutant and keeping other parameters constant (using 25 ml of 100 ppm pollutant at room temperature). Figure 14 showed that the adsorption was rapid about 80% in the first 30 min, then, by increasing time from 30 to 240 min the adsorption capacity increased to about 95% after which the adsorption capacity remained constant indicating that it reached equilibrium^73^.

**Figure 15:**
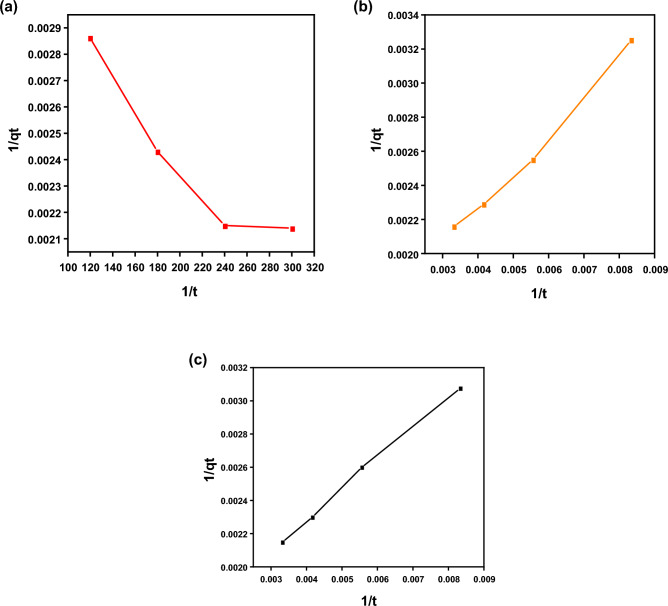
(**a**) Pseudo-1st-order for E122 adsorption, (**b**) Pseudo-1st-order for E110 adsorption, and (**c**) Pseudo-1st-order for Cr(VI) adsorption.

**Figure 16:**
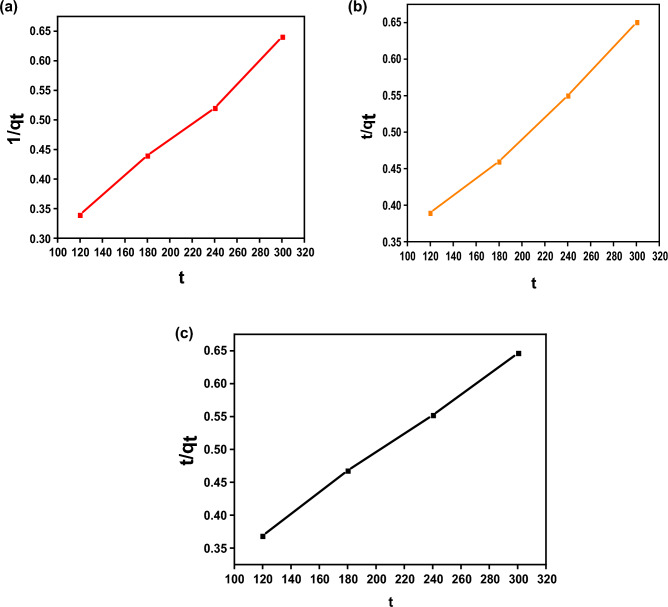
(**a**) Pseudo-2nd-order for E122 adsorption, (**b**) Pseudo-2nd-order for E110 adsorption, and (**c**) Pseudo-2nd-order for Cr(VI) adsorption.

Adsorption kinetic models can determine the adsorption mechanism and even the physicochemical properties of the adsorbent^74^. Therefore, two models, pseudo-second-order and pseudo-first-order, were employed to investigate the adsorption of E110, E122 and Cr(VI). The kinetic models were explained in their nonlinear form, as illustrated in Figs. 15 and 16. The attained parameters of each model are given in Table 4. The pseudo-second-order and pseudo-first-order models frequently reveal the surface-controlled adsorption process. In the present study, the pseudo-second-order model fits better than the pseudo-first-order model where the correlation coefficient (R^2^) is very close to unity (Table 4). Therefore, the adsorption of E110, E122 and Cr(VI) on the surface of CS@CTAB is controlled by a chemisorption mechanism.

**Table 4:** Kinetic parameters for (E122, E110 and Cr(VI)) adsorption on to CS@CTAB.

Sorbents	k_1_ (min^-1^)	q_e1_(mg/g)	q_e_ (Experimental) (mg/g)	R^2^
Pseudo-1st-order				
CS@CTAB -E122	96.59	632.9	466	0.96862
CS@CTAB- E110	160.09	724.64	461.54	0.98652
CS@CTAB- Cr(VI)	120.64	649.35	464.75	0.99644

Sorbents	k_2_(g/(mg.min))	q_e2_(mg/g)	q_e_ (Experimental) (mg/g)	R^2^
Pseudo-2nd-order				
CS@CTAB- E122	1.87 × 10^–5^	613.5	465	0.9913
CS@CTAB- E110	1.011 × 10^–5^	689.65	461.54	0.99094
CS@CTAB-Cr(VI)	1.24 × 10^–5^	653.6	464.75	0.99866

#### Effect of temperature and thermodynamic studies

**Figure 17:**
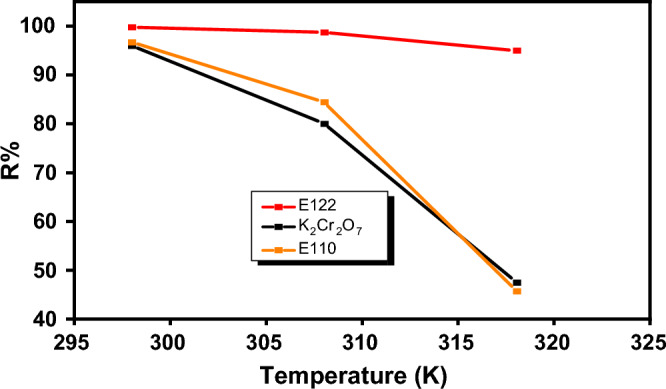
Effect of temperature on E110, E122, and Cr(VI) adsorption on to CS@CTAB adsorbent.

To examine temperature influence on the adsorption capacity of CS@CTAB, isotherms were attained in the range of (298-318 K). Equilibrium isotherms for investigated temperatures were demonstrated in Fig. 17. From Fig. 17, it was noted that the adsorption capacity on CS@CTAB decreased by increasing temperature^31^.

To explore the adsorption process of Cr(VI), E110, and E122 dyes onto the CS@CTAB surface in terms of spontaneity and feasibility and to determine the degree of randomness at the solid/liquid interface, adsorption thermodynamic parameters were determined.

The adsorption of Cr(VI), E110, and E122 was studied at different temperatures from 298 to 318 K at pH 2 for Cr(VI) and pH3 for the E110 and E122 for 4 h. Free energy parameter (ΔG^o^ads), adsorption entropy parameter (ΔS^◦^ads), and heat of enthalpy parameter (ΔH^◦^ads) of Cr(VI) metal ion, and E110 and E122 dyes adsorption by CS@CTAB adsorbent were calculated. ΔG^o^ads parameter was calculated from the following Eqs. (9) and (10).

**Figure 18:**
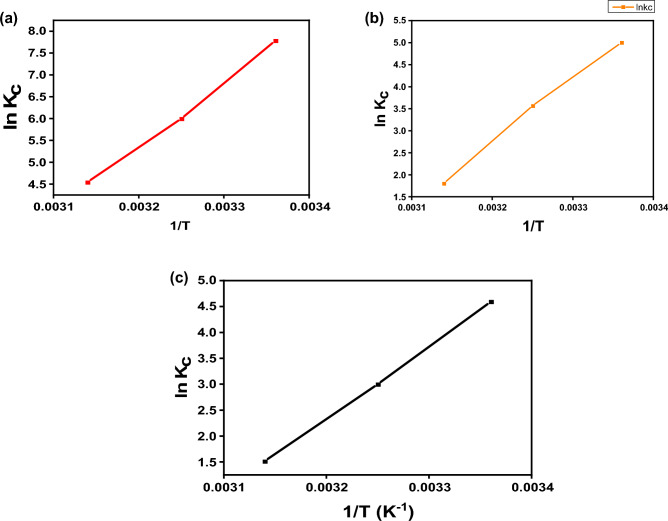
Plot of ln K_C_ versus (1/T) absolute temperature for the adsorption of (**a**) E122, (**b**) E110, and (**c**) Cr(VI) on to CS@CTAB.

From the experimental data that present in Table 5, it was noticed that the negative ΔG^o^adsn value which confirms that the adsorption of Cr(VI), E110 and E122 by CS@CTAB adsorbent is a spontaneous process. It was also observed that the negative ΔH^o^ads value confirms that the adsorption of Cr(VI), E110, and E122 by CS@CTAB material is exothermic. From ΔH^o^ values that are higher than 80kJ/mole, it was proven that the adsorption was chemisorption. The negative ΔS^o^ads values showed that Cr(VI) metal ion, E110, and E122 adsorption onto CS@CTAB surface leads to lower disorder and higher arrangement^37^. The plot of lnK_C_ versus(1/T) absolute temperature for the adsorption of E122, E110 and Cr(VI) onto CS@CTAB is shown in Fig. 18.

**Table 5:** Thermodynamic parameters for the E122, E110 and Cr(VI) adsorption on to CS@CTAB:

System	K_c_			∆G(KJ/mol)			∆S° (J/mol. K)	∆H° (KJ/mol)
	298 K	308 K	318 K	298 K	308 K	318 K		
CS@CTAB- E122	2392.27	403.429	95	− 19.28	− 15.36	− 12.03	− 122.06	− 345.91
CS@CTAB- E110	245	35.52	6.11	− 12.388	− 9.142	− 4.785	− 120.553	− 363.031
CS@CTAB- Cr(VI)	98.73	20.086	4.55	− 11.372	− 7.68	− 4.019	− 116.02	− 351.81

#### Effect of ionic strength

**Figure 19:**
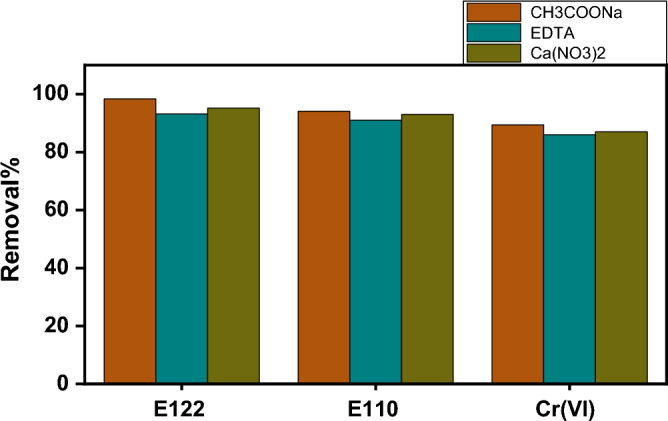
Effect of ionic strength on E110, E122, and Cr(VI) adsorption on CS@CTAB adsorbent.

For the investigation of the ionic strength effect several species have been used. This procedure is crucial due to the presence of adverse ions in industrial wastewater with high concentrations. So, to study the effect of ionic strength the following species are used: CH_3_COONa, EDTA, and Ca(NO_3_)_2_ at optimum adsorption parameters. The influence of different anions on the adsorption of E110, E122, and Cr(VI) was presented in Fig. 19. The effect of different anions having a concentration of 0.1 M is insignificant on the adsorption of pollutants under investigation.

#### Regeneration experiments

**Figure 20:**
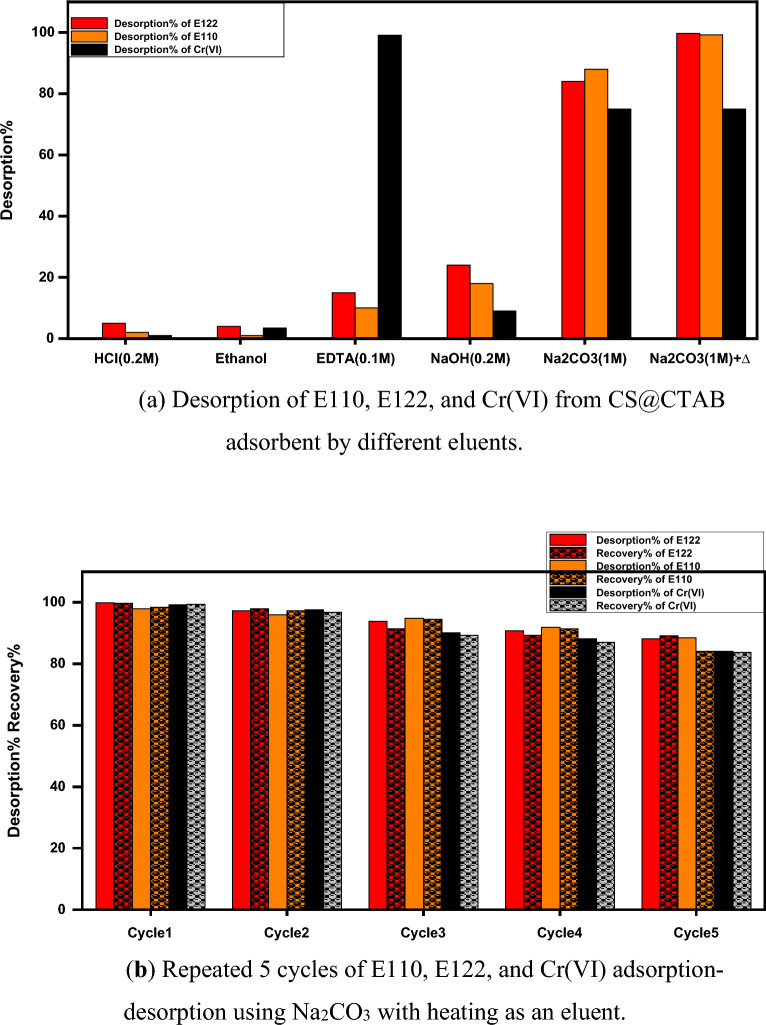
(**a**) Desorption of E110, E122, and Cr(VI) from CS@CTAB adsorbent by different eluents. (**b**) Repeated 5 cycles of E110, E122, and Cr(VI) adsorption–desorption using Na_2_CO_3_ with heating as an eluent.

To test the reusability of CS@CTAB, the desorption procedure was performed under optimum parameters. Subsequently, we examined the result of applying adverse eluents like HCl, NaOH, ethanol, and Na_2_CO_3_ (with and without heating) as presented in Fig. 20a and it was observed that the effect of Na_2_CO_3_ with heating at 45 °C achieved the best results for the desorption of E122, while Na_2_CO_3_ alone was the best eluent for E110 and eventually EDTA presented the best desorption for Cr(VI). Hence HCl, ethanol, and NaOH are poorly affecting the desorption of the three investigated pollutants. Figure 20b shows the influence of Na_2_CO_3_ at room temperature, Na_2_CO_3_ at 45 °C, and EDTA eluents on the desorption of E110, E122, and Cr(VI), respectively through five repeated cycles of adsorption–desorption^75^.

#### Removal of different pollutants from binary systems

**Figure 21:**
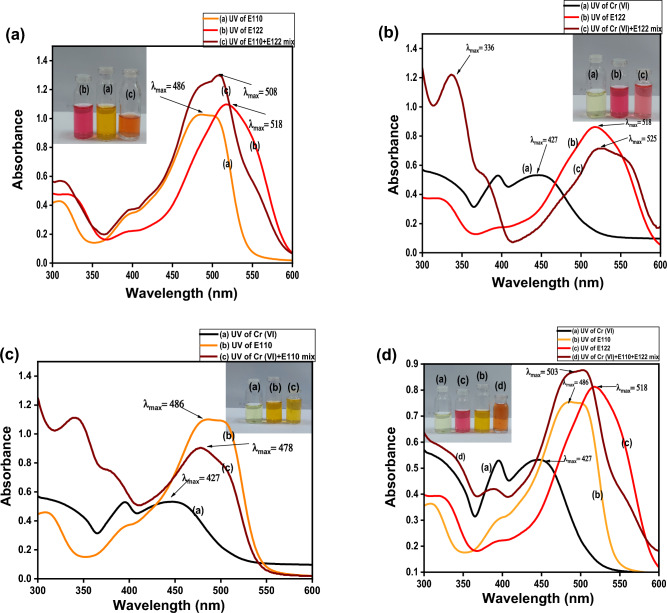
UV data of (**a**) E110 + E122 mix is compared with E110 and E122, (**b**) Cr(VI) + E122 mix is compared with Cr(VI) and E122, (**c**) Cr(VI) + E110 mix is compared with Cr(VI) and E110, and (**d**) E110-E122 + Cr(VI) mix is compared with E110, E122, and Cr(VI).

**Figure 22:**
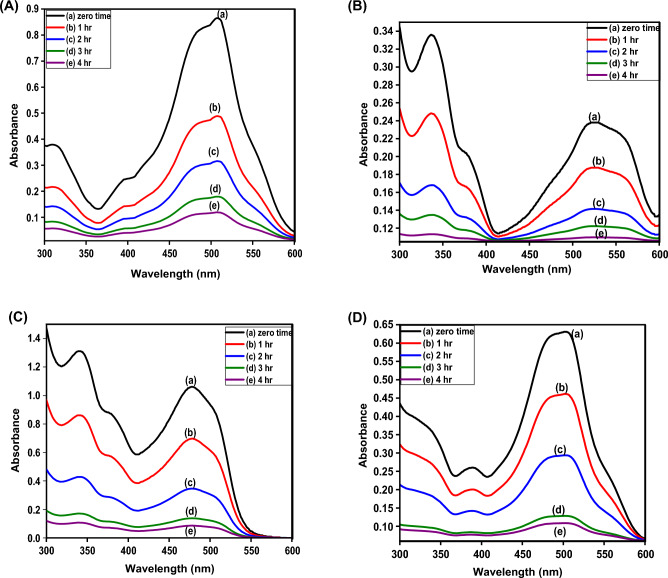
UV data of after adsorption by CS@CTAB at different periods of time: (**a**) E110 + E122 mix, (**b**) Cr(VI) + E122 mix, (**c**) Cr(VI) + E110 mix, and (**d**) E110-E122 + Cr(VI) mix.

To examine the effect of CS@CTAB on the removal efficiency of pollutants in binary systems, the λ_max_ for each pollutant and that for each binary system were measured as follows: E110 (486 nm), E122 (518 nm), and Cr(VI) (427 nm), while λ_max_ of binary systems are: E110 + E122 (508 nm), E110 + Cr(VI) (478 nm), E122 + Cr(VI) (525 nm), and E110 + E122-Cr(VI) (505 nm). New overlapped peaks appeared after the formation of binary systems then adsorption studies took place at each new λ_max_ appeared. Adsorption took place by applying 0.005 g of CS@CTAB in 25 ml of binary system solutions with 50 ppm of each pollutant to form the binary system at pH 3, then undergoes shaking at 120 rpm. Maximum adsorption of binary systems took place after 4 h as shown in UV–visible data in Figs. 21 and 22. The adsorption efficiency of E110, E122, and Cr(VI) was estimated from Eq. (2).

#### Application

##### In natural water samples

To assess the effectiveness of CS@CTAB for the adsorption of different pollutants, ideal experimental circumstances were fitted to natural samples. Standard solutions were used to create calibration curves. Under the above-optimized experimental circumstances, standard solutions (1.0 L) were handled. Several water samples, including tap water from our lab, and seawater from Ras Elbar City, Egypt, were used as analytical samples. Table 6 displays the analysis outcomes. The recoveries of spiked samples with a known amount of each pollutant were investigated. The recovered amounts ranged from 96.00 to 99.69%. These findings suggest that the CS@CTAB can be used to accurately to identify E110, E122, and Cr(VI) in natural water samples.

**Table 6:** Analytical results of adsorption of E110, Cr(VI) and E122 in natural water samples employing CS@CTAB as an adsorbent. (n = 5).

Sample	Pollutant	Spiked (µg/mL)	Measured (µg/mL)	Recovered (µg/mL)	Recovery (%)
Tap water	E110	0.00	0.00	0.00	0.00
		50	2.01	47.99	99.69
		100	5.61	94.39	98.047
	E122	0.00	0.00	0.00	0.00
		50	1.43	48.57	99.122
		100	3.08	96.92	98.89
	Cr(VI)	0.00	0.00	0.00	0.00
		50	1.32	48.68	99.65
		100	5.07	94.93	97.16
Sea water	E110	0.00	0.00	0.00	0.00
		50	3.1	46.9	97.43
		100	6.87	93.13	96.74
	E122	0.00	0.00	0.00	0.00
		50	1.98	48.02	98
		100	3.8	96.2	97.85
	Cr(VI)	0.00	0.00	0.00	0.00
		50	2.1	47.9	98.06
		100	6.06	93.94	96.15

##### In colored soft drinks and food industrial

**Figure 23:**
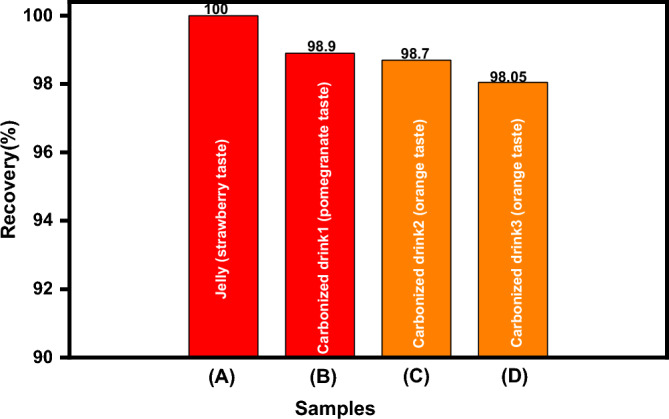
Recovery (%) of 100 ppm E122 from (**A**) strawberry tasted jelly, (**B**) pomegranate carbonized drink1 and 100 ppm E110 from (**C**), and (**D**) two different orange carbonized drinks.

To assess the CS@CTAB performance for anionic dye adsorption, different samples containing E110 and E122 were subjected to the optimum experimental factors for E110 and E122 adsorption. The standard solutions were used to create the calibration curves. Degassed carbonated beverages and jelly made up the food samples. Figure 23 shows that more than 98% of the E110 and E122 extraction from the tested samples was accomplished. These findings suggest that the removal of E122 and E110 from various samples could be accomplished using the CS@CTAB adsorbent^76,77^.

#### Reasonable mechanism of Cr(VI), E110, and E122 adsorption onto CS@CTAB

To investigate the possible mechanism of metal ion adsorption onto CS@CTAB, the morphology, surface charge, optical images and FTIR of the adsorbent were evaluated.

**Figure 24:**
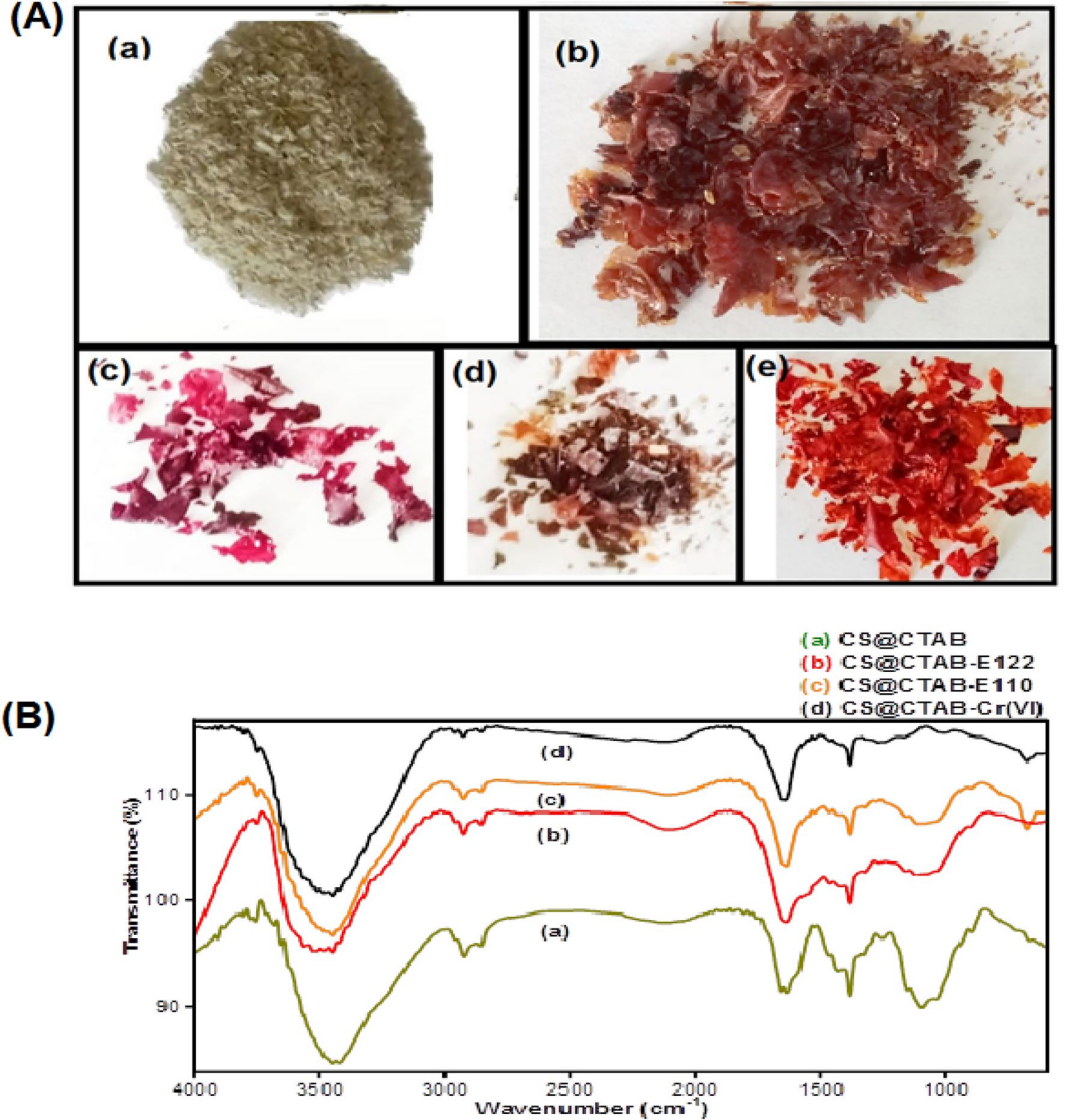
**(A)** Digital photographs of (**a**) Chitosan (CS) (**b**) CS@CTAB (**c**) CS@CTAB-E122 (**d**) CS@CTAB-Cr(VI) (**e**) CS@CTAB-E110. **(B)** IR spectra of (**a**) CS@CTAB, (**b**) CS@CTAB-E122, and (**c**) CS@CTAB -E110, (**d**) CS@CTAB-Cr(VI).

Optical images of the chitosan, CS@CTAB, CS@CTAB-E122, CS@CTAB-Cr(VI), and CS@CTAB-E110 are represented in Fig. 24A(a–e). A great change appeared in the color of the chitosan, CS@CTAB, CS@CTAB-E122, CS@CTAB-Cr(VI), and CS@CTAB-E110. The beige color of chitosan (Fig. 24A.a) changed to brown in CTAB-modified chitosan (CS@CTAB) after the reaction of chitosan with CTAB in the presence of glutaraldehyde (Fig. 24A.b). After adsorption of each pollutant, the color of the CS@CTAB was changed from brown to pink in the adsorption of E122 (Fig. 24A.c), to yellow in the adsorption of Cr(VI) (Fig. 24A.d), and to orange in the adsorption of E110 (Fig. 24A.e). Those outcomes show the adsorption ability of CS@CTAB towards the pollutants.

The FT-IR spectra of CS@CTAB after loading of E122, E110, and Cr(VI) are shown in Fig. 24B A change in the stretching vibration of the hydroxyl group can be observed in the IR spectra of CS@CTAB-Cr(VI), CS@CTAB-E122, and CS@CTAB-E110 as the peak of the OH group of CS@CTAB undergoes a slight shift as the peaks at 3683 cm^−1^ and 3462 cm^−1^ are shifted to 3750 cm^−1^ and 3518 cm^−1^ at CS@CTAB-Cr(VI), CS@CTAB-E122, and CS@CTAB-E110, and the inhomogeneous broadening of the peak after adsorption indicates the formation of medium H-bonding^78^. Additionally, the fork shape of the (N–H) bending peak at 1650 cm^−1^ disappeared in the IR spectra of CS@CTAB-Cr(VI), CS@CTAB-E122, and CS@CTAB-E110, indicating its reaction. In the IR spectra of CS@CTAB-E122 and CS@CTAB-E110, new peaks appeared at 1315 cm^−1^ and 1075 cm^−1^, attributed to (S–O) bending vibration and strong (S=O) stretching of the (SO_3_) group. On top of a shift in (C–O) bending vibration from 1155 to 1162 cm^−1^ in the IR spectra of both CS@CTAB-E122 and CS@CTAB-E110^79–82^.

**Figure 25:**
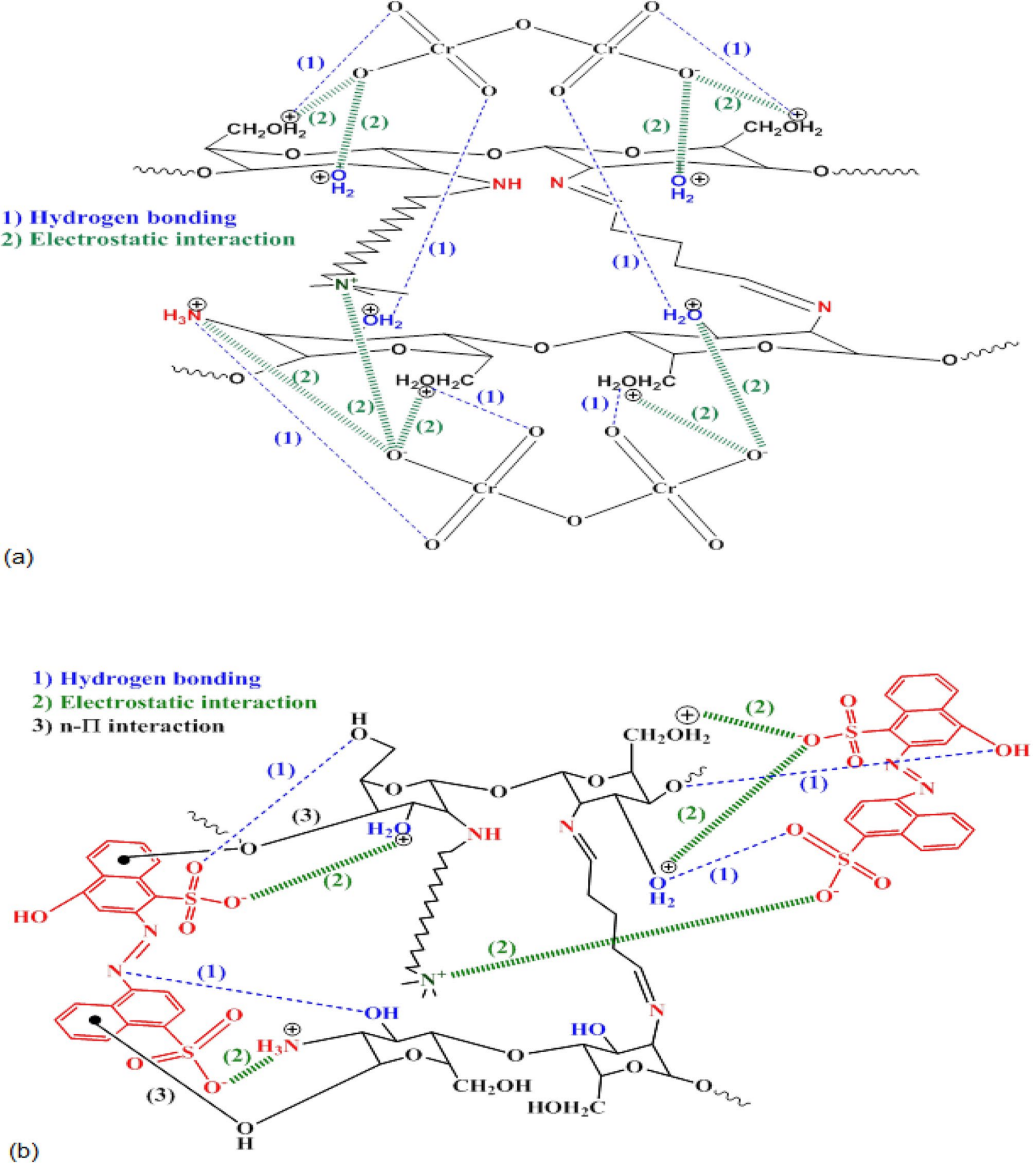
Resonable mechanism of adsorption of (**a**) Cr(VI) ions and (**b**) anionic dyes (E122 and E110) onto CS@CTAB.

The adsorption mechanism of the investigated anionic species was predicted by taking into consideration the functional groups on the surface of CS@CTAB, as shown in Fig. 25a,b. These functional groups include amino (-NH_2_) and hydroxyl (–OH) groups. As illustrated in Fig. 25a, For Cr(VI), in an acidic medium, the CS@CTAB adsorbs Cr(VI) through the electrostatic interactions between the negatively charged oxygen on the Cr(VI) and the positively charged groups (–NH_3_^+^, –OH_2_^+^) of the CS@CTAB adsorbent. Furthermore, hydrogen bonding has a vital role in the adsorption process for the three anionic species via the interaction between hydrogen on the surface of the adsorbent and atoms including oxygen and nitrogen in the structure of the anionic species, respectively^83^. Eventually, Fig. 25b illustrates the n-π's interactions are also attributed to the adsorption of the anionic dyes (E122 and E110) by the interaction of the electron-donating groups, which can be presented by the nitrogen and oxygen groups of the adsorbent and the aromatic rings of both dyes^84^.

#### Performance of CS@CTAB

Table 7 demonstrates the performance of CS@CTAB in comparison with other adsorbents. As noted from comparison studies, several variables should be taken into consideration, for example, adsorption dose, adsorption capacity, equilibration time, the sorbent type, and the initially applied concentration. From the studies, CS@CTAB proved to have a higher capacity towards Cr(VI), E122, and E110, as represented in Table 7.

**Table 7:** Comparison of adsorption capacity of E122, E110 and K_2_Cr_2_O_7_ onto CS@CTAB with previously reported studies.

Adsorbate	Adsorbent	Adsorbent dose	Initial concentration	Equilibrium time	Adsorption capacity (mg/g)	References
E122	Doped (Ag) ZnO _NPS_	0.08 g	5 ppm	80 min	84.7	^9^
	ND	1 g/L	50 ppm	60 min	12	^11^
	0.1SnO_2_–0.9CeO_2_	1 g/L	20 ppm	20 min	22.10	^69^
	0.15SnO_2_–0.85CeO_2_				22.88	
	0.2SnO_2_–0.8CeO_2_				21.16	
	0.3SnO_2_–0.7CeO_2_				18.33	
	CS@CTAB	0.005 g/25 ml	100 ppm	240 min	490	Present study
E110	PDMAEMA grafted microspheres	0.4 g	20 ppm	40 min	312.5	^53^
	CTS-AP	1.5 g	50 ppm	60 min	95	^85^
	(Fe_3_O_4_-EA) in MSPE	100 mg/25 mL	1 µg/L	5 min	45	^86^
	CPC modified silica	0.4 g/25 mL	4 × 10^–5^ mol/L	15 min	5.14 μmol/g	^87^
	CS@CTAB	0.005 g/25 ml	100 ppm	240 min	481.35	Present study
K_2_Cr_2_O_7_	PPy/MoS_2_	0.015 g/50 mL	50 ppm	24 h	257.73	^88^
	Chitosan/g/C_3_N_4_/TiO_2_	0.33 g	100 ppm	24 h	165.3	^19^
	Coffee ground and mixed waste tea	5 g	100 ppm	120 min	94.34 (coffee ground)	^89^
					87.72 (Mixed waste tea)	
	A-RS/PVA	0.01 g/20 mL	100 ppm	48 h	140.39	^90^
	CS@CTAB	0.005 g/25 ml	100 ppm	240 min	488.5	Present study

## Conclusion

CS@CTAB was fabricated via facile methodology followed by adsorption of these anionic species (E122, E110, and Cr(VI)). The CS@CTAB adsorbent contains a functional quaternary ammonium group, providing a large number of active adsorption sites. Hence, this adsorbent shows convenient recycling properties and notable practicality. We noted an efficient role for CS@CTAB in the adsorption of sunset yellow FCF, Azorubine, and hexavalent chromium (individually and in combination) at the optimum conditions of 0.005 g dose, 100 ppm initial concentration, and 240 min for all pollutants, while a pH of 3 was used for both dyes and 2 for Cr(VI). The adsorption kinetics of anionic pollutants on the surface of CS@CTAB was well-fitted to the pseudo-second-order model, and the isotherms were in agreement with the Langmuir isotherm with a maximum adsorption capacity of 492.6 mg/g, 492.6 mg/g, and 490.196 mg/g for Azorubine, Sunset Yellow, and hexavalent chromium, respectively. Thermodynamic studies suggest that the adsorption procedure is considered exothermic and spontaneous. From the previous findings, the adsorption of the CS@CTAB for anionic species was mono-layer chemisorption. Eventually, the eco-friendly and biodegradable CS@CTAB will be an attractive candidate for removing anionic species from aquatic systems and industrial samples.

## Data Availability

All data generated or analyzed during this study are included in this published article.
